# A policy response to workplace innovation for the rail sector

**DOI:** 10.12688/openreseurope.13933.2

**Published:** 2021-11-08

**Authors:** GARAZI CARRANZA, Oihane De la Rua, Begoña Sanchez

**Affiliations:** 1 Mafex-Spanish Railway Association, Bizkaia, Spain; 2 Tecnalia Research and Innovation, Bizkaia, Spain

**Keywords:** Workplace Innovation, Open Innovation, rail, small and medium enterprises

## Abstract

The rail sector is a sector with a significant impact on European industry and it is therefore important that it follows the current innovative trends. We live in an increasingly digitised society but, until now, digitisation has not been a priority issue for the sector as the rules that apply to the entire value chain have hindered the digitisation process. Even so, technologies are not enough, and innovation must be implemented in companies at the organisational and employee level. The RailActivation project has experimented with workplace innovation to foster innovation capabilities in the railway sector, providing elements for companies to remain as innovative and competitive as possible, as well as to have additional tools to adapt to these challenges. In order to help in this process, this article proposes a series of recommendations based on the lessons learnt during the implementation of the project. These recommendations establish a link between policy and workplace innovation practices and could be a reference framework for further research and policy. The suggested policy recommendations are focused on companies and policy makers and are based on the results obtained from the different consultations with the stakeholders involved in this research.

## Plain language summary

Businesses face daily challenges as a result of an increasingly globalised economy. Rapid technological advances, changing demographics and rapidly evolving consumer demands require companies to constantly reinvent themselves in order to survive, and to do so, management teams need the support of the entire workforce. ‘Workplace innovation’ (WI) defines organisational practices that enable employees at different levels to use and develop their skills, knowledge, experience, and creativity to the fullest, in order to improve the company's performance as well as their own well-being. The RailActivation project has experimented with workplace innovation in the railway sector to keep companies competitive by providing them with the tools they need to adapt to the new challenges in the best possible way. This article develops a series of recommendations, for both companies and policy makers, that are intended to serve as a reference for future research in the field of workplace innovation as well as to help companies and policy makers who intend to implement related actions and programmes. 

## Introduction

The rail sector is synonymous of technology, efficiency, and sustainability, being one of the most energy-efficient transport modes, responsible for 9% of passenger and 7% of freight transport ( Tattini & Teter, 2020). The railway industry is an important sector for Europe, with a turnover of 49,2 billion Euros and an expected annual growth rate of 2,3% until 2025 ( Schwilling, 2020).

The sector has experienced annual growth of 3,6% since 2017, largely driven by significant investments in rolling stock, infrastructure and rail control. In addition, the number of vehicles and amount of track kilometres in operation has grown significantly ( Schwilling, 2020).

The COVID-19 crisis has brought about rapid and disruptive changes, putting digitalisation at the core. The railway sector is a slowly evolving sector, and the needs identified have highlighted the need to accelerate innovation processes, both technological and non-technological, to put digitalisation at the heart of the industry. Non-technological aspects will play an important role as much as technological aspects, as they are necessary to foster innovation capabilities that allow companies to continue innovating in order to adapt to changes more quickly and smoothly. In addition, technologies and non-technological aspects of innovation will also play a vital role in the organisational culture of companies, as a new revolution in the demand for supplies is taking place for which companies must be prepared.

RailActivation ( http://railactivation.eu/the-project/) aims to help in the solution of this problem, and its main objective is to create and pilot an organisational mechanism for the railway sector that will help in the adoption of innovation in the workplace, with special emphasis on small and medium enterprises (SMEs) in the railway sector, in order to create an open innovation ecosystem. Improving innovation services and technological development will be more important than ever, and workplace innovation (WI) will play a vital role on that process.

According to the European Commission (EC) if companies want to remain competitive, they must invest in both technological and non-technological innovation and WI presents an opportunity for this. However, rail sector innovation is focus on technological developments to adapt to market requirements, therefore WI has not been the priority for the sector up to date. RailActivation will develop new mechanisms and tools to anticipate the effects of digitalisation in the railway sector and design the transformation of the innovation process for the EU railway industry, working from the inside of companies to the outside, with the customer at the heart of the activities and enabling workers to be part of the change through WI. According to several studies, workplace innovation improves employees’ motivation as well as offering better working conditions. This translates into an increase of the productivity at work, a greater capacity to develop innovation, a better resilience to changes in the marker and a greater competitiveness of the company. The European Workplace Innovation Network (EUWIN) initiative evidenced that the productivity of entities applying WI has been 20-60% ( Totterdill *et al.*, 2016) higher than in the ones implementing traditional methods.

The rail industry, especially SMEs, need to fast track their technological innovation in order to remain competitive in the digital revolution. Technologies are not enough for this; innovation needs to be applied in companies at both the organizational and employees’ levels. RailActivation project tests WI in railway SMEs to foster innovation capacities, providing adapted methodologies for companies to remain as innovative and competitive as possible as well as to have additional tools to adapt to these challenges. To achieve this, RailActivation has developed and tested a pilot scheme based on WI, as all enterprises should be aware of the benefits that WI brings for their organizations. WI should be part of the company´s business models and strategies. For this reason, the objectives of the project include the following: raise awareness and dissemination of WI, and the creation of an inter-regional networks to assist in the process.

The recommendations presented in this report summarize the results obtained by the RailActivation Project, which has received funding from the European Union’s Horizon 2020 research and innovation program. The project starts by investigating and analysing the key competences and skills that presently characterize the railway manufacturing sector ( Carranza *et al.*, 2020); as a state of the art. RailActivation is a pioneer project that has managed to pilot test WI techniques within the rail sector, a sector that lacks from innovation. The number of stakeholders reached with the project by the different campaigns and interactions show the impact that RailActivation has had. The pilot scheme outlined has proven to be a very good tool for companies from the sector to implement WI and could be a reference for other sectors as it could be easily transferred. The expected impacts of the project are the creation of a new context based on the support mechanisms created for WI in SMEs, more SMEs benefiting from the opportunities created by the WI, the creation of new forms of innovation in the workplace, improved conditions for implementing new technologies, and a more skilled workforce and more resilient enterprises.

The project is carried out by an international consortium formed by: MAFEX- Spanish Railway Association ( https://www.mafex.es/en/), TECNALIA Research & Innovation ( https://www.tecnalia.com/en/), DITECFER ( https://www.ditecfer.eu/en/), Rail.S ( https://rail-s.de/), and QUINN—Consorzio Universitario Qualità e Innovazione ( https://www.consorzioquinn.it/en/homepage/). This paper provides policy recommendations to establish a link between WI policies and practices. These recommendations are a framework of reference for additional research as well as for companies and policy makers to put in place additional specific measures and programs that could support the identified needs.

## Theoretical background

Workplace innovation is a new and emerging concept ( Kesselring *et al.*, 2014) that combines structural and cultural practices in order to increase the involvement of employees in the changes taking place in the company as well as in the organisational renewal of it. Within workplace innovations agendas such as innovation, digitalisation, productivity, job quality, lifelong learning, wellbeing at work, skills, and social dialogue are integrated ( Pot *et al.*, 2020).

WI is a comprehensive approach to create fair working. The key principles to achieving this are defined in the European Pillar of Social Rights act (‘Fair Working Conditions’) ( European Commission, 2021) and are innovative forms of work that ensure quality working conditions are to be promoted, social dialogue, and employee participation, a high level of protection of the employees’ health and safety at work and environment which is adapted to the professional needs of the employees which also enables them to prolong their participation in the labour market ( Pot *et al.*, 2020).

According to the European Commission, workplace innovation improves the performance and working life of employees and serves as a tool for fostering workers’ creativity through organisational change. It seeks to combine leadership with the practical knowledge gained by frontline workers, thus involving the different parts of the process in the changes. Innovation in the workplace is based on open dialogue between different parts involved, not everyone in the process has the same knowledge, experience and learning, and sharing results in the creation of new ways of working through participatory actions ( Kesselring *et al.*, 2014).

WI results are evidenced by many studies. According to Totterdil *et al.*, if we analyse the “European workers percentage involved in improving work organization or processes this numbers are not really high (47%), neither the percentage of consulted employees before setting the targets for their work (47%)” ( Totterdill *et al.*, 2016). Further studies verify that companies gain competitive advantage when applying technical innovation and/or organisational innovation through their entities ( Evangelista & Vezzani, 2010). Another advantage that has been seen is that if the organisation provides its employees with autonomy at work, the possibility to work in a teams, the opportunity to consult others, on-the-job learning, etc., the risk of psychological stress is reduced ( Pot, 2017). According to Oeij *et al.* (2016) the drivers for WI implementation are divided into two main groups: The first group focuses on improving the economic objectives of the organisation and on a better quality through an increase in productivity, a better quality of the products manufactured or a better customer service, for example. The second group focuses more on the quality of the employees' working life, as well as their commitment to the company and the work they perform. The increase in employee motivation and well-being are key factors in reducing stress at work, improving job satisfaction and mental health, among other factors ( Oeij *et al*., 2016).

There is a need to raise awareness among the management of organisations of the benefits of workplace innovation, as very few organisations have WI in the structure of the organisation. That’s why the European Commission should keep on supporting the specific and combined roles of social partners, professional organisations and researchers in scaling up evidence-based practices and as well as the operation of learning networks as a mean of disseminating and resourcing WI ( Pot *et al.*, 2020). Since social partnerships can contribute to the upward convergence of working conditions by improving the implementation and functioning of policies at EU, Member State, sectoral, and organisational levels.

The EU rail sector has a set of particularities and it requires a specific approach to maximize effectiveness of WI implementation. Railway sector has focused their competitiveness on technological innovation. According to Garazi Carranza *et al.,* “The influence of combined organizational factors and individual employee behaviour adoption has not been thoroughly analysed in the railway sector. However, research among European firms indicates a positive relation between non-technological innovation and organizational performance, all resulting in more dynamism, innovation capacity and competitiveness” ( Carranza *et al.*, 2020).

## Methods

The recommendations are based on the feedback gained from companies during the RailActivation pilot, especially from SMEs and other type of organisations that have been consulted ( Carranza *et al.*, 2020). Below we define the activities that served to test the Pilot Scheme as already indicated and the policy response to WI:

- **Rail live event 1-2 ^nd^ December 2020**. Big dissemination event where the project and the Pilot Scheme where presented. Feedback was also gathered from participants and especially from companies for project follow up and for recommendations.
- **Innovation Way® Workshops on 10 ^th^ and 11 ^th^ December 2020**. Workplace Innovation was explained, and pilot tested with participant companies. The Pilot Scheme was also presented and tested. A survey by means of Slido was made to companies participating to get feedback on Workplace Innovation, Pilot Scheme, and recommendations for further action among others.
- **Slido pool prepared for the Innovation Way® Workshops**. Companies were asked on their needs regarding WI as well as on the Pilot Scheme and potential needs for further recommendations.
- **Workshop on 16 ^th^ February 2021**, oriented to awareness and dissemination on Workplace Innovation as well as on the RailActivation Pilot Scheme. Valuable feedback was gathered from the participants.
- **Additional Survey launched on 18 ^th^ February 2021**. Results have served for the concluding remarks as well as for the recommendations suggested.
- **Continuous follow up and test of project results especially with the Clusters partners of RailActivation project**. Very relevant feedback obtained on continuous basis from the interaction with companies.
- **External feedback** obtained by means of an **EU survey launched** aimed at assessing awareness, operational knowledge and practices on Workplace Innovation in European organisations made from **12th November 15th to December, 2020**. Results from this survey have been also considered. The Survey ran online (on Google forms: https://forms.gle/DcSLFfw21eAyDhNUA) from 12th November to 15th December, 2020. 36 organizations from 12 H2020 Countries participated: Belgium (1), Denmark (1), France (2), Germany (6), Ireland (1), Italy (7), Moldova (3), Serbia (2), Spain (9), Sweden (1), Turkey (1), and United Kingdom (2). The participants included: Cluster organizations (66,7%), Industry associations (5,6%), Trade Unions (2,8%), Development Agencies (2,8%), Others, including universities, innovation centres, business accelerators, and SMEs (22,2%).
- **Additional Dissemination activities** where we informally have also gathered feedback on the project related activities.

Firstly, a mapping phase was carried out in which an EU analysis and a benchmarking analysis were included, with the aim of identifying the possible SMEs and stakeholders that had strong interest and motivation of joining the RailActivation project. As part of the mapping phase, the analysis of practices, tools, mechanisms, and schemes were analysed, permitting to identify the challenges for WI practices. The analysis was made by an online questionnaire covering a wide range of public opinion in railway sector. The survey was drawn out based on the results of the benchmark carried out and common European workplace innovation concept and indicators ( Kesselring *et al.*, 2014). The survey contains 34 questions divided in four sections: Employee level, organizational level, process level, and results level, which cover the different layers of workplace innovation proposed by Workplace innovation: Concepts and indicators. Data was collected over a 54-day period (between 02/12/2019 and 24/01/2020) and the final sample included 203 respondents from 16 European countries ( Carranza *et al.*, 2020). No inclusion or exclusion criteria was applied, as this was an anonymous survey to obtain information and feedback. Based on the analysis and literature review, a new WI pilot scheme for railway industry is defined based on implementing the open culture and improving productivity of the sector ( http://railactivation.eu/documents-2/). 

**Figure 1.**
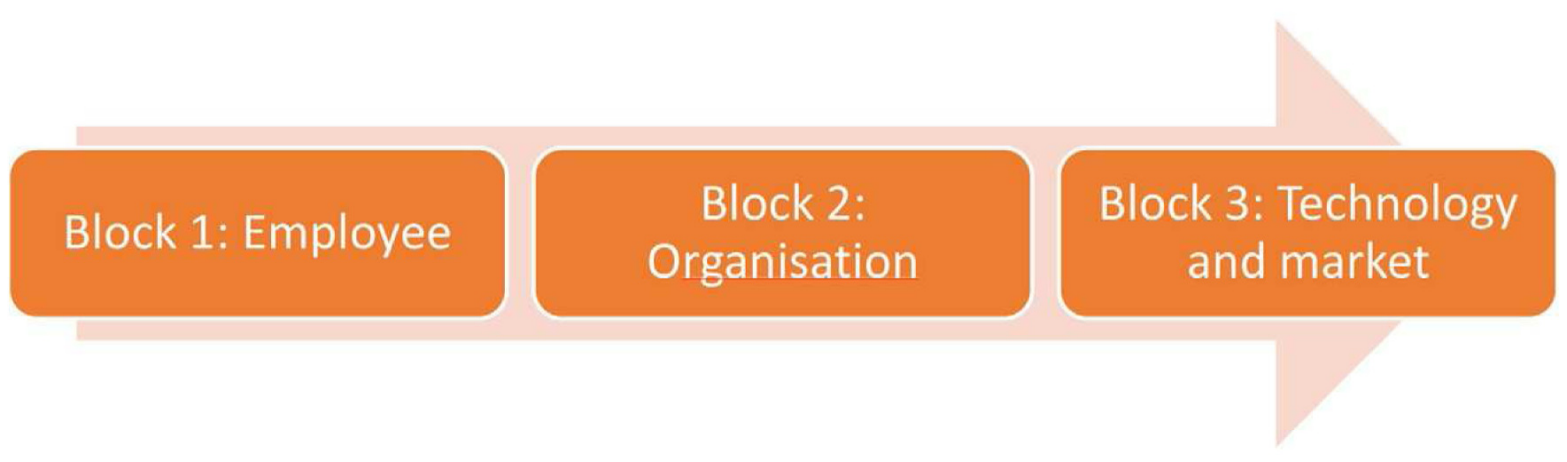
RailActivation scheme blocks.

The scheme was conceived as an Itinerary for companies, especially SMEs, to understand where they stand with WI. The RailActivation pilot scheme is proposed as a flexible itinerary where the company can check and jump on the specific block they need to improve. Furthermore, the companies can select the blocks based on their specific needs (see Figure 1). This means that some companies will go for all the blocks while others will select and implement the ones they want to improve.

The WI scheme drives the company through an itinerary to understand where they stand on WI focusing on the following three interrelated aspects: organization, employees and technology and market. The pilot scheme has been tested over a 10-month period, and from it results recommendations have been developed to boost WI in rail sector ( http://railactivation.eu/documents-2/).

In order to define methodology, information from 32 SMEs were collected: 10 German, 10 Italian and 12 Spanish. With the aim of testing the WI methodology for rail sector three editions of four workshops were organised, involving 18 SMES and 55 employees. These workshops added to the ‘individual’ learning an additional element generating value for the participants: the sharing and co-creation of ideas. This was done through teamwork sessions and through the shared presentation of results ( http://railactivation.eu/documents-2/). The obstacles that emerged before acceptance of the proposed approach were the involvement of technical profiles focused mainly on product innovation, reduced recognition of the value of communication and management, and focus on tasks less on getting a complete overview of the company.

In order to analyse in detail the degree of knowledge about WI of European innovation intermediaries, an additional survey was launched from November 12th to December 15th, in which 36 organizations from 12 countries participated. From this analysis the need to support the implementation of WI in companies is confirmed. The analysis done during the project shown 30,6% of participants didn’t know about WI before the RailActivation project and 54,4% of participants had never organized initiatives on WI.

Furthermore, a Slido poll was prepared to collect responses to the workshops from participants on the pilot scheme and to get feedback to prepare the policy recommendations report ( http://railactivation.eu/documents-2/).

## Results

Half of the participants consider the employee block from the pilot scheme as the most important aspect of the scheme, followed by the organisational block, and then the technology and market improvement ( Carranza *et al.*, 2020). This means the entities priorities are focused on better engagement and involvement of employees, followed by advancing towards a more strategic and smarter organisation. Thus, the entities do not focus their innovation on looking for new technologies though market and customers to improve their own products and services. At the employee level, 83% participants confirm the importance of feeling engaged in the innovation process of the company. Additionally, the analysis confirms training is also highlighted as a very important aspect (67%).

At the organizational level, more than a half of the participants considered teamwork as important aspect in the workplace (58%). In this sense, the communication methodologies gain importance, being considered as a strategic aspect to improve WI (58%).

At the technology level, less than a half of the participants consider the need for continuous review and update the technology used internally, being the technology innovation mainly focused on product and service development (33%).

Based on the results obtained in the WI workshops the policy recommendations are defined. The methodology for the delivered recommendations consisted of three editions of workshops with four workshops in each of them, in which 107 people participated across all workshops. 

## Policy response towards WI

The policy recommendations intend to establish a link between WI policies and practices. These recommendations could be also a framework of reference for additional research as well as for policy makers to put in place additional specific measures and programmes that could support the identified needs. The recommendations suggested target two main target groups.

- Regulators and policy makers as well as relevant agencies facilitating the implementation of key enabling technologies, transformation towards innovation models, and service innovation.
- Actors from railway value chain and operators, especially companies and society in general.

By examining the evidence gained during the implementation of the project, the links between WI policies and competitiveness of the SMEs are highlighted. 

## Recommendations addressing companies

The following recommendations are suggested for companies based on the work developed with them:

### R1. Promotion of employee-centred actions

The results described in the methods section confirm that the employee block is the most important one in WI, followed by organization and technology/market. As a result, actions where employee’s engagement is promoted need to be supported. For implementing WI, organizations must move towards a more strategic and organizational structure. This means employees should be more involved in the tasks and in the decision-making process. When it comes to technology and market improvement, innovation in this area should come by the search of new technologies, new markets and customers, and the improvement of the existing products and services.

Technology is closely linked to a company's competitiveness and productivity; for this reason, it is very important to know how to use it properly and to keep it up to date with the constant updates it undergoes. Technology refers to the development of new methods/practices that result from the application of different WI instruments. To keep them up to date, companies should invest more in marketing actions and in analysing the behaviour of markets and the needs of consumers. Also, in order to keep abreast of the emerging new technologies, the companies should have a specific department focused on technology, which ensures that new technologies are continuously analysed and applied in the company. In this sense, companies should constantly review their own technologies and update them when necessary. Staying up to date in this area brings great benefits to companies, such as more optimised production processes, increased productivity, better communication, reduced costs, better decision making, and a more competitive company. It is also important to consider technology as a key aspect for WI as it has been shown that, by involving it, products are developed faster and more cost-effectively.

Internal communication is also an aspect to be taken into account, and it is important to keep in mind the technologies present in this area as good internal communication serves as a support for agile and innovative collaboration within the company. In addition, information dissemination channels should be made available so that information is transmitted in a more fluid way, both inside and outside the company and so that it is accessible at all times to whoever needs it.

### R2. Foster innovation in the workplace through a clearer, more flexible, participative and innovative work structure

Companies need an organisational structure that meets the minimum requirements in terms of clarity, economy, company vision, employee understanding of tasks, decision-making, company adaptability, maintenance and innovation. Such structure enables employees and managers to engage in WI.

For a successful innovation process in the workplace, it is important for employees to be able to express themselves clearly. Often employees are afraid to express themselves because of reprimands or because the hierarchical system of the company does not make easy for them to do so. This is why companies should use systems to improve in this aspect, for example through regular feedback, greater transparency, reaching out to employees in a more proactive way, not highlighting the leader's position so much, or providing feedbacks on actions that arose from suggestions or criticisms made by employees.

In addition, it has been found that employee involvement in decision making leads to higher productivity, as employees' active participation in various aspects of the company leads to greater effort on their part, as they want their efforts to succeed overall. This is beneficial for both the company and the employees themselves, as the employees are indirectly forming and preparing themselves for greater responsibilities in the future. Moreover, having employees recognised for their work leads to a good atmosphere and friendship in the workplace; this allows employees to feel that they can be authentic without fear of being treated differently or punished. As a consequence, the company's performance increases.

It is essential that the company enables all its employees to understand the tasks they have to carry out and that they have clearly identified their responsibilities. It is necessary that all employees of the company understand how their tasks fit into the overall mission of the company, so that they can relate their efforts to the common mission of the company. In turn, they need to know how to imply the mission of the organisation in their own tasks, contributions or directions.

**Figure 2.**
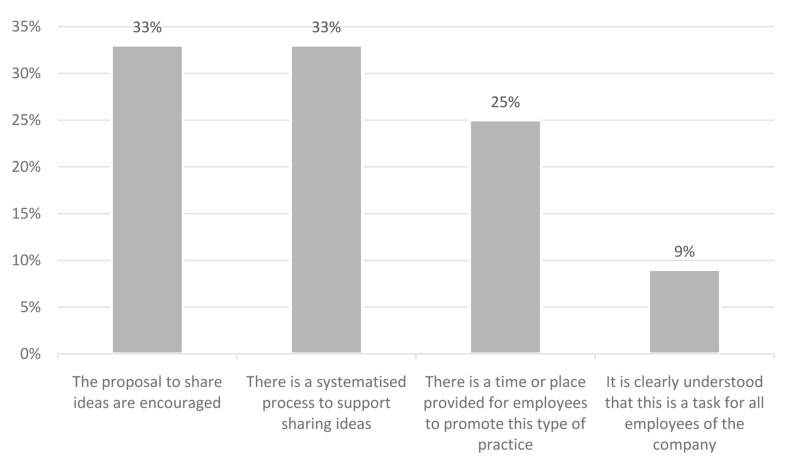
Sharing of ideas in the companies.

Among the results obtained from the project, it has also been seen that employees value the fact of having means to share ideas with each other. It is necessary to establish an open dialogue between employees to achieve a smarter organisation, with better working conditions and more time for personal development that will be of great benefit to the company, in terms of improvements and services. As it can be seen in the Figure 2 below, many companies in the pilot already had methods in place to encourage communication in this sense. Greater interaction between employees can be achieved through brainstorming sessions, team building, rooms dedicated to collaboration or interaction between employees, among others, or even having a space for breaks where employees from different departments can interact. Sharing knowledge enhances employee creativity, fosters innovation, and improves results, at the individual, team, and organisational levels.

The following chart shows the results:

It has also been seen that all of the above can be fostered or developed through the increase of innovation in the workplace, but in order to successfully develop WI, it is important to use new practices to organise internal procedures. The most relevant aspect is the use of new practices to organise internal procedures, such as supply-side change management, business re-engineering knowledge management, lean production, quality management, etc.

### R3: Innovation culture is a necessity for all companies and a mechanism to ensure it should be promoted

Companies should invest time in promoting a more innovative culture by generating new ideas and implementing them. When the company works in an innovative ecosystem, benefits increase, employees are more committed to their work, and the overall health of the company improves.

For this the transparency of the company is an important aspect to take into account. Information about the company must be available for consultation, the company must provide it voluntarily, and it must be useful, clear, verifiable, and truthful. Employees, customers, shareholders, or suppliers have more confidence in companies that are more transparent.

Furthermore, it has been seen that for a greater innovative culture it is important to promote cross-functional teamwork: work groups formed by members belonging to different departments. Different points of views from employees need to be taken into account from technical to marketing, etc. The sharing of information between the different departments of the company provides different points of view that help in the realisation of projects and in the resolution of problems, as the members of the group can contribute with different solutions or recommendations, this can lead to better impacts and inputs for the company and a better analysis of all the paths which will lead to an increase in competitiveness.

People are born creative and this is something that companies need to take advantage of. A company whose philosophy supports the creativity of employees will constantly be innovating. To do this, it is necessary to provide employees with methods to share their ideas with others, as the different points of view of employees can lead to the creation of new ideas that benefit the company. But to keep employees motivated in this respect, it is important that they receive feedback on the contributions they have made. Rewarding employees can also increase their motivation.

### R4. Matching training and capacity building with the company´s needs

Companies should support their employees in continuous training. This brings benefits to both the company and the employees themselves as it increases satisfaction, motivation, and participation among workers. Workers have a better knowledge of the tasks they carry out, which increases their safety and leads to higher productivity and fewer accidents at work. In addition, a better trained worker is a better candidate for promotion, which improves his or her working conditions.

This type of training can be carried out by different methods, such as professional masters, continuous vocational training, seminars, workshops, conferences, professional development courses, among others.

### R5. Communication policies/strategies should be prioritised both internally and externally of the companies

On the one hand, with regards to the internal communication of the company, from the obtained results it has been seen that employees highly valued the fact of having methods to disseminate internally within the company, for example through a centralised system where employees can have access to the information they need at all time. In addition, it is important that the content of the information is adapted, so that all employees are able to understand it. Furthermore, a common language is needed to innovate within the company; the lack of a common language hinders coordination between different levels of authority and across departments and this slows down innovation, which is why the development of a common language is something that needs to be worked internally within the company. On the other hand, in terms of the company's communication with the outside world, it has been found that sharing information with other companies or institutions can bring great benefits to companies, as it helps to exchange opinions and enables companies to have better vision of the cooperation strategy. Cooperation with other companies to develop products/processes has been highlighted by companies during the RailActivation project implementation actions (described in the methods section), especially for innovation as well as for other type of sources such as for attending and participating in conferences, trade fairs, etc.

### R6. Companies should have a clear strategy for cooperation

Co-operation with other companies or institutions to develop products/processes can be beneficial for companies especially in the area of innovation. The collaborating companies may have different business models that can bring advantages to the other company, but for that the companies must be willing to adapt their business model to changes or adaptations as appropriate. In addition, one of the companies may have experiences to share about previous projects at the regional, national, or European level, which may be useful if the other company is developing something in that field. 

In order to develop this collaborative culture, it is important for companies to attend collaborative events more regularly, to participate actively in trade fairs and conferences and to work actively within the stakeholder group to which they are related to.

### R7: Gender should be considered to ensure Workplace Innovation success

Diversity in the workplace brings great benefits to companies. When work groups are made up of different genders, people with different skills, tastes and personalities, the results are much more enriching.

Among the surveyed companies, only 8% of senior management positions were held by women. 33% stated that there was a differentiation between the work performed by men and women.

According to Esteve-Volart, gender discrimination in companies leads to less entrepreneurial talent and a slower accumulation of female human capital, which leads to slower adoption of technologies, a less innovative company, and lower economic growth ( Cuberes & Teignier, 2016). Where gender equality exists, the results have been found to be more positive than without it, increasing the company's productivity capacity and economic performance ( Klasen & Minasyan, 2017) ( Morais Maceira, 2017).

## Recommendations addressing policy makers

Firstly, policies that address uncertainty are needed. The uncertainty and complexity generated by the COVID-19 crisis forces the rail industry's strategy and management to be readjusted. In the mid-term, we could foresee a transformation in the strategic and operational models. It is true that before the pandemic, disruptive elements were already on the table that invited us to rethink on supply chains (Ecosystems 4.0, robotization, reindustrialization, etc.) but COVID-19 has served as a catalyst for a paradigm shift. In this sense, for example, the European Commission has recently launched the European Innovation Council (EIC) with a budget of over €10 billion for the years 2021–2027 to develop and expand breakthrough innovation, via projects as the new Horizon Europe program which combines research on emerging technologies with an accelerator program and a dedicated equity fund to scale up innovative start-ups and SMEs ( European Commission, 2020). 

Secondly, funding from private entities needs to increase, especially in aspects related to innovation. The COVID-19 crisis has highlighted the need to accelerate innovation processes, both technological and non-technological innovations, in order to ensure that industry has digitalisation at its core. In order to face this situation, the organizational culture of the rail industry must be prepared for a new revolution that will be based on technologies focused on adapting the demand to supply requirements. In 2014, Venture Capital (VC) investment in the European Union was around €5 billion, while in the United States this amount was much higher, reaching €26 billion. In terms of venture capital funds, the United States was again well ahead of the European with a sum of €120 million for EEUU and €60 million for Europe ( Europe, n.d.). Private equities and venture capitals investments help on the creation of more successful businesses with stronger and more sustainable futures.

Thirdly, policies related to regulations and innovation, nowadays, there are not many companies that implement a model capable of measuring the return made in digitalization. We could say, therefore, that the big challenge for the sector is to provide innovative products that offer flexibility to respond to changing market demands. In this sense, the technological innovation must be implemented together with non-technological innovation, in which WI represents an opportunity. The European Commission, in addition to providing companies with financial support, should also offer them subsequent advice, since in some sectors such as transport, regulations are very complex, in order to help developers to better understand the regulatory framework that applies to them. Innovation Deals for example seek to assist in the identification and addressing of the perceived legislative obstacles by providing a greater clarity on the existing legislations and the identification of the possible solutions, through the involvement of both national and European national authorities and the different regulators. ( Bogers *et al.,* 2018).

And finally, it has been seen that there is a need to create an inter-regional network for the railway sector to serve as a mechanism to foster innovation in the workplace. WI is not yet a priority for European companies and the vast majority of European companies will need a first step before they can create an inter-regional network for knowledge sharing. The RailActivation scheme has proved to be a very useful tool to help companies in the railway sector during the WI implementation process and thus to have an impact on the performance of the railway industry directly or indirectly. The RailActivation scheme has enabled a better understanding of specific barriers to innovation, competences, cooperation, and investment, summarising the results of feedback collected from SMEs active in the clusters of the partnership. Although developed around companies in the railway sector, the scheme is expected to be transferable to companies from different sectors. Furthermore, during the Innovation Way ^®^ Workshops it has been found that the scheme works best in manufacturing companies with 100–200 employees, manufacturing companies with around 10 employees, and IT companies with 10–20 employees.

After carrying out the project, it has become clear that there is a need in the railway sector for more research into the effects of WI programs, several European countries have a good record of achievement in incentivising, assigning and sustaining resources for workplace innovation. Yet there is not any existing programme or experience in the field of workplace innovation. From a methodological point of view, it has been found that the best results are obtained through a combination of surveys and case studies.

More indepth research will be needed to understand the barriers organizations face when considering workplace innovations and whether there are effective combinations of interventions.

The recommendations will be presented to different institutions to send impact messages for a better positioning of the railway sector on the needs of SMEs in the sector.

The year 2021 being the European Year of Rail ( https://europa.eu/year-of-rail/index_es) will serve as a driving action, as further support to the rail sector is expected through EU promoted initiatives that will ensure the European Green Deal ( https://ec.europa.eu/info/strategy/priorities-2019-2024/european-green-deal_en) and the Sustainable Mobility Strategy ( https://ec.europa.eu/transport/themes/mobilitystrategy_en) through the implementation of technology and innovation.

## Discussion

At the centre of transitions are often leaders and working groups that have the ability to think more openly and consequently have an enhanced ability to create long-term perspectives. The COVID-19 pandemic brought important changes that affect consumer behaviour, supply chains, and, therefore, the competitiveness of the rail industry. In this context, the sector faces the challenge of aligning the supply chain with market, in which they must incorporate elements such as advanced planning and digitalization. Based on the literature review, it can be seen that the culture of innovation, the high levels of employee engagement and the transformational capacity of both organisations and individuals have been influential in the development of digital transformation. The digital transformation is a key element for companies to remain competitive in a market that is constantly changing, and which is also becoming more and more demanding.

However, to succeed in the technological transformation of rail, a culture that promotes innovation and creativity is needed. While technological investment is increasing, the digital revolution presents other needs to successfully complete the transformation ( Carranza *et al.*, 2020). In this sense, adapting the values, procedures and experiences that define the organisation through its employees is one of the biggest challenges in the digital era ( Carranza *et al.*, 2020). WI is not yet a concept that the companies know as such, but this does not mean that they do not consider it important neither that they are not implementing it. In this situation, workplace innovation will play an important role, especially for SMEs, in terms of the development of innovative services and the technological development of companies. Experience-based learning and investment in risk-minimising technologies can benefit companies that want to be smarter and more flexible.

Therefore, it can be concluded that a successful change in the work system has to be combined with an increase of employees involvement. In addition, it is important that employees are committed to an innovative culture and that they are up to date and have the necessary information available to develop their personal competences, through which they will help the company to evolve.

In view of the obtained results, it has been found that more research is needed to better understand the way on how European policy, national programs, and initiatives promote WI.

This is especially relevant for the railway sector, where WI provides a valuable resource grappling with the challenge of boosting the sustainable mobility of Europe. More studies and research projects will be welcomed to better understand the situation and the specific measures needed to support it, especially during 2021, as the European Year of Rail.

As can be see, WI is not mainstream yet, and European clusters and ecosystems’ stakeholders need a preparatory step before being able to create an interregional network for knowledge.

Thanks to the WI scheme proposed in the RailActivation project, and after testing it with the different companies involved in the project, it will be possible to better understand the specific barriers to innovation, skills, cooperation, and investment in the railway sector. Toward this end, to ensure best practices, identification and exchange should be promoted at EU level using WI mechanisms and tools applicable to the railway SMEs needs.

Furthermore, to increase the impact and EU added value, the creation of synergies with other projects and countries is a key tool. Collaboration across regional and national boundaries has proven to be an important mean of improving both the effectiveness and efficiency of policies and programs.

There is a need to continue supporting the railway sector by means of new measures that will permit to accelerate the technological and innovation adoption and implementation in the sector in view of guaranteeing the Green Deal strategy as well as sustainable mobility for the railway sector.

## Conclusion

Future research on the preconditions for participatory structures within work teams will be necessary. This is an essential part of workplace innovation as it is directly related to the innovative behavior of workers and the commitment they show towards the company. Companies need workers who are committed to what they do and to the company itself, as well as showing a disposition to deal with the industrial revolution affecting the railway sector. Rail transport plays a key role for Europe’s economy and society but has the potential to contribute much more. The sector is experiencing both a severe skills shortage and a need for re/upskilling. A large share of workforce is expected to retire in next 10 years, while the job attractiveness should increase. In turn, the major transformation process driven by research and innovation requires to bring in suitable skills and competencies. In addition, further research on COVID-19 related issues affecting long-term rail performance is needed to promote strategies to ensure the long-term sustainability of the rail sector.

## Data availability

All data underlying the results are available as part of the article and no additional source data are required.
