# Supplementary material for: A policy response to workplace innovation for the rail sector
Source: Open Res Eur. 2021 Nov 8;1:108. Originally published 2021 Sep 14. [Version 2] doi: 10.12688/openreseurope.13933.2 (PMC10445933; doi:10.12688/openreseurope.13933.2)
Supplement: Peer review history

# Peer review: Reviewer response for version 2

**DOI:** 10.21956/openreseurope.15385.r28012

**Published:** 2021-11-30

**Authors:** Vassil Kirov

**Affiliations:** 1 Institute of philosophy and sociology, Bulgarian academy of sciences, Bulgarian academy of sciences, Sofia, Bulgaria

The revisions made by the authors are appropriate.

Are sufficient details provided to allow replication of the method development and its use by others?

Partly

Is the description of the method technically sound?

Yes

Are the conclusions about the method and its performance adequately supported by the findings presented in the article?

Partly

Is the rationale for developing the new method (or application) clearly explained

Yes

If any results are presented, are all the source data underlying the results available to ensure full reproducibility?

Yes

Reviewer Expertise:

NA

I confirm that I have read this submission and believe that I have an appropriate level of expertise to confirm that it is of an acceptable scientific standard.

# Peer review: Reviewer response for version 1

**DOI:** 10.21956/openreseurope.15017.r27766

**Published:** 2021-10-22

**Authors:** José Antonio Casado

**Affiliations:** 1 Education, Research, Innovation and Knowledge Transfer, University of Cantabria, Santander, Spain

The article begins with a state of the art of the railway sector and a definition of Workplace Innovation. This section defines the needs of the sector and how WI will help to address these needs.

 In terms of the methodology followed in the RailActivation project, the format followed in running the workshops using the Innovation Way methodology is outlined. For this purpose, the three blocks into which they have been separated are presented, marking the objectives of each one and the three steps to be followed during the implementation of the Pilot Scheme, in which the steps followed and the material used for some parts of the analysis are shown.

 Finally, the conclusions summarise the results obtained in the project, based on what was previously presented.

Are sufficient details provided to allow replication of the method development and its use by others?

Yes

Is the description of the method technically sound?

Yes

Are the conclusions about the method and its performance adequately supported by the findings presented in the article?

Yes

Is the rationale for developing the new method (or application) clearly explained

Yes

If any results are presented, are all the source data underlying the results available to ensure full reproducibility?

Yes

Reviewer Expertise:

Mechanical and microstructural chatacterization of railway superstructure components

I confirm that I have read this submission and believe that I have an appropriate level of expertise to confirm that it is of an acceptable scientific standard.

# Peer review: Reviewer response for version 1

**DOI:** 10.21956/openreseurope.15017.r27604

**Published:** 2021-10-15

**Authors:** Vassil Kirov

**Affiliations:** 1 Institute of philosophy and sociology, Bulgarian academy of sciences, Bulgarian academy of sciences, Sofia, Bulgaria

The proposed article is based on the workplace related experimentation in the framework of the EU H2020 project RaiActivation. In general the article is convincing even if there are some statements that are not sufficiently explained - e.g. not sure why "The COVID-19 crisis has highlighted the need to accelerate innovation processes, both technological and non-technological innovations, in order to ensure that industry has digitalisation at its core".

 The argument is partially related to future actions, so I'm wondering to what extent it would not be stronger if published at a later stage of the project development. 

 The theoretical background is based on the workplace innovation concept and I agree with the authors that this concept should be tailored according to the specifics of the sectors, in this case of the railway sector. The methodology seems appropriate, but some additional explanations would be useful to allow reproducibility. The results are presented in a very concise way and are not discussed sufficiently. The recommendations, based on the analysis are well developed, convincing and could have for sure a practical effect, the only minor concern is about the eventual national specifics in relation to workplace innovation. The conclusion could be further developed. There are some important elements, introduced only at the level of the conclusions - "A large share of workforce is expected to retire in next 10 years, while the job attractiveness should

 increase". 

 In general the article is interesting and valuable but should be further developed, according to the above-mentioned comments.

Are sufficient details provided to allow replication of the method development and its use by others?

Partly

Is the description of the method technically sound?

Yes

Are the conclusions about the method and its performance adequately supported by the findings presented in the article?

Partly

Is the rationale for developing the new method (or application) clearly explained

Yes

If any results are presented, are all the source data underlying the results available to ensure full reproducibility?

Yes

Reviewer Expertise:

Sociology of work and organisations

I confirm that I have read this submission and believe that I have an appropriate level of expertise to confirm that it is of an acceptable scientific standard, however I have significant reservations, as outlined above.

# Response

**Published:** 2021-10-28

**Authors:** GARAZI CARRANZA

Dear Reviewer, 

 Thank you very much for your comments. We have updated the new version according to your comments. the methodology explained is the article is the methodology the EU funded project has implemented to achieve the results. Therefore, unfortunately, this methodology is defined in the way it was implemented and cannot be modified. We will take the comments for future studies.
